# The histamine H_4_ receptor: from orphan to the clinic

**DOI:** 10.3389/fphar.2015.00065

**Published:** 2015-03-31

**Authors:** Robin L. Thurmond

**Affiliations:** Janssen Research & Development, LLCSan Diego, CA, USA

**Keywords:** histamine, atopic dermatitis, pruritus, asthma, arthritis

## Abstract

The histamine H_4_ receptor (H_4_R) was first noted as a sequence in genomic databases that had features of a class A G-protein coupled receptor. This putative receptor was found to bind histamine consistent with its homology to other histamine receptors and thus became the fourth member of the histamine receptor family. Due to the previous success of drugs that target the H_1_ and H_2_ receptors, an effort was made to understand the function of this new receptor and determine if it represented a viable drug target. Taking advantage of the vast literature on the function of histamine, a search for histamine activity that did not appear to be mediated by the other three histamine receptors was undertaken. From this asthma and pruritus emerged as areas of particular interest. Histamine has long been suspected to play a role in the pathogenesis of asthma, but antihistamines that target the H_1_ and H_2_ receptors have not been shown to be effective for this condition. The use of selective ligands in animal models of asthma has now potentially filled this gap by showing a role for the H_4_R in mediating lung function and inflammation. A similar story exists for chronic pruritus associated with conditions such as atopic dermatitis. Antihistamines that target the H_1_ receptor are effective in reducing acute pruritus, but are ineffective in pruritus experienced by patients with atopic dermatitis. As for asthma, animal models have now suggested a role for the H_4_R in mediating pruritic responses, with antagonists of the H_4_R reducing pruritus in a number of different conditions. The anti-pruritic effect of H_4_R antagonists has recently been shown in human clinical studies, validating the preclinical findings in the animal models. A selective H_4_R antagonist inhibited histamine-induced pruritus in health volunteers and reduced pruritus in patients with atopic dermatitis. The history to date of the H_4_R provides an excellent example of the deorphanization of a novel receptor and the translation of this into clinical efficacy in humans.

## Introduction

There are now four known G-coupled protein receptors (GPCRs) that use histamine as a ligand. These receptors were discovered over a span of almost 100 years and each discovery provides excellent examples of the use of state-of-the-art receptor pharmacology to discovery new receptors. The first actions of histamine were noted around 1910 (Barger and Dale, 1910; Dale and Laidlaw, 1911), but this before the idea of receptors was widely accepted. After the development of compounds that blocked the effect of histamine in the 1930–1940s, it was noted that their effects were consistent with a competition for binding at a receptor now known as the histamine H_1_ receptor (H_1_R; Wells et al., 1945). These first antihistamines became the basis for very successful drugs, some of which are still in use today. However, they also revealed new questions since there were actions of histamine that were not blocked by these ligands. This led to the proposal that a second histamine receptor existed (Ash and Schild, 1966), and the discovery of selective ligands for this receptor led to its pharmacological characterization and the designation as the H_2_R (Black et al., 1972). A similar story exist for the discovery of the H_3_R, where it was noted that various histamine receptor ligands modulated histamine actions in the brain, but that the pharmacology did not match the known H_1_R and H_2_R (Arrang et al., 1983).

## Discovery of the Histamine H_**4**_ Receptor

However, by the 1990s novel receptors were discovered mainly by the identification of their gene sequence and less so by their pharmacology. The cloning of the receptor cDNA for the human H_1_R and H_2_R were described in the early 1990s (Gantz et al., 1991; De Backer et al., 1993), but the sequence of the H_3_R remained elusive despite much work looking for sequences with similarity to the H_1_R and H_2_R. The breakthrough came using a different approach where sequences were identified that contained general homologies to GPCR (e.g., predicted seven transmembrane domains and other common residues). These putative orphan GPCR genes were then screened for binding to likely ligands. Using such an approach, a novel sequence was discovered that encoded a GPCR that bound to histamine and had pharmacology that matched that described for the H_3_R (Lovenberg et al., 1999). The details of the sequence revealed why the gene for the H_3_R was not found by a homology approach, since it only exhibited ∼20% homology to the H_1_R and H_2_R.

While the cloning of the H_3_R used an orphan GPCR/reverse pharmacology approach, it yielded a receptor that was already known and characterized on a pharmacological basis. The discovery of the H_4_R truly started with the identification of an orphan GPCR. The discovery of the gene for the H_3_R provided a tool for additional homology searches of databases for related sequences. This yielded a putative receptor that was ∼35% identical to the H_3_R and when expressed in heterologous systems was found to have a high affinity for histamine (Liu et al., 2001). These genomic approaches were so prevalent at the time that the identification of this new receptor, now known as the histamine H_4_ receptor (H_4_R), was described almost simultaneously by six different laboratories (Nakamura et al., 2000; Oda et al., 2000; Liu et al., 2001; Morse et al., 2001; Nguyen et al., 2001; Zhu et al., 2001). Unlike the H_3_R, the H_4_R was completely novel and its function was unknown. However, one hint of function was evident from the expression pattern described in the original cloning papers that showed a fairly selective expression on bone marrow and hematopoietic cells known to be involved in inflammatory and immune responses.

## Development of Selective Ligands

The identification of a novel histamine receptor, its expression mainly on cells involved in immune responses coupled with the successful history of drugs that target the H_1_R and H_2_R generated immediate interest (Hough, 2001). However, deorphanizing the receptor was only the beginning. Importantly, selective ligands were needed to be able to understand the function of the receptor. The receptor has a relatively high homology to the H_3_R and thus it is not surprising that many of the compounds previously described as H_3_R ligands also had affinity for the H_4_R (Liu et al., 2001). These ligands included (R)-α-methylhistamine, an H_3_R agonist, and thioperamide, an H_3_R antagonists, but because these ligands had affinity for both receptors they are not ideal tools for understanding the function of the H_4_R. The first potent and selective H_4_R antagonist was JNJ 7777120 (Jablonowski et al., 2003). This compound has a high affinity for the H_4_R and is highly selective relative to other histamine receptors (Thurmond et al., 2004).

JNJ 7777120 has become an excellent tool for understanding the physiological role of the H_4_R and has been used extensively *in vitro* cell models and in animal models. The use of JNJ 7777120 *in vivo* provided the first evidence that H_4_R antagonists could have anti-inflammatory properties. Neutrophil influx in a mouse peritonitis model was reduced upon pretreatment with JNJ 7777120 (Thurmond et al., 2004). Early work also showed that JNJ 7777120 and its analog, JNJ 10191584, were also efficacious in a rat colitis model (Varga et al., 2005). This compound along with other H_4_R antagonists have shown activity in models of asthma, dermatitis, pain, and pruritus among others (**Table 1**; Dunford et al., 2006, 2007; Cowden et al., 2010b; Hsieh et al., 2010).

**Table 1:** Effects of H_**4**_R antagonists in selected animal models^**1**^.

Disease models	Effect	Reference
Asthma	Decreased BAL eosinophils and lymphocytes Decreased IL-5, IL-4, IFNγ, IL-17, PGD_2_, LTB_4,_ and TNF levels Decreased lung T cells, CCL3, CCL5, mucin, and collagen Increased lipocortin-1 Decreased antigen specific IgE and IgG_1_ levels Decreased airway inflammation Decreased cough and dyspnea Decreased lung resistance, tissue stiffness, and tissue dampening	Dunford et al. (2006), Cowden et al. (2010a), Beermann et al. (2012), Somma et al. (2013), Thurmond et al. (2014a)
Dermatitis	In acute FITC dermatitis model reduced inflammation, skin eosinophils and mast cells, IL-4, IL-6, TNF, MIP1α, IL-1β, MCP-1, GM-CSF, RANTES, and KC levels No effect in other acute hapten models or in dog In chronic models reduced skin lesions, skin eosinophils and mast cells, IgE, IL-4, IL-5, IL-6, TSLP, TARC, and NGF levels	Rossbach et al. (2009), Cowden et al. (2010b), Seike et al. (2010), Baeumer et al. (2011), Suwa et al. (2011), Matsushita et al. (2012), Ohsawa and Hirasawa (2012), Thurmond et al. (2014a)
Pruritus	Reduced histamine-induced scratching Reduced scratching in acute hapten models Reduced scratching in chronic hapten models	Dunford et al. (2007), Cowart et al. (2008), Cowden et al. (2010b), Liu et al. (2008), Rossbach et al. (2009), Yamaura et al. (2009), Koenig et al. (2010), Suwa et al. (2011), Ohsawa and Hirasawa (2012), Shin et al. (2012), Savall et al. (2014)
EAE	Increased clinical score, demyelination, and inflammation Increased IFNγ and IL-17 Reduced IL-4 and IL-10	Ballerini et al. (2013)
Arthritis	Decreased severity score Decreased inflammation, pannus formation, cartilage and bone damage Decreased Th17 cells and IL-17 production	Cowden et al. (2014)
Pain	Reduced inflammatory, neuropathic, and post-operative pain	Cowart et al. (2008), Liu et al. (2008), Hsieh et al. (2010)
Colitis	Reduced lesion area Reduced tissue TNF and neutrophil levels	Varga et al. (2005)


^1^BAL, bronchoalveolar lavage; EAE, experimental autoimmune encephalomyelitis.

As evidenced by the pharmacology of the other histamine receptors, it is important not to rely on the data provided by a single ligand when assigning function to a particular receptor. The reason for this is that despite the best efforts it is almost impossible to completely understand the pharmacology of any ligand and how it may differ in different species, cell types, or conditions. Because of these factors, researchers must use a combination of tools including agonist/antagonist pairs, ligands from different chemical classes, and/or knock-out animals before making conclusions about the function of receptor. The use of a single ligand only gives firm conclusions about the action of that ligand under the given experimental conditions.

This has been highlighted as a potential issue with JNJ 7777120 as it has been described as having agonistic effects at the H_4_R of some species in some, but not all, transfected cell models and has been described to drive functional selectivity, i.e., arrestin activation, of the H_4_R (Seifert et al., 2011). However, while these other pharmacological effects may exist, most of the current data support the fact that JNJ 7777120 is an antagonist *in vivo* and in primary cells. For example the effects on JNJ 7777120 in models of asthma, dermatitis, arthritis, pain, and peritonitis are consistent to those of other distinct H_4_R ligands and in H_4_R-deficient mice (**Table 2**; Thurmond et al., 2004, 2014a; Dunford et al., 2006; Coruzzi et al., 2007; Altenbach et al., 2008; Cowart et al., 2008; Liu et al., 2008; Cowden et al., 2010b, 2014; Hsieh et al., 2010; Shin et al., 2012; Savall et al., 2014). Perhaps one of the best examples of this is in the role of the receptor in mediating pruritic responses in mice. Dunford et al. (2007) reported that histamine-induced scratching in mice could be blocked by JNJ 77777120 and did not occur in H_4_R-deficient mice. Furthermore, other H_4_R agonists also induce scratching that can be blocked by JNJ 7777120, but cannot induce scratching in H_4_R-deficient mice (Dunford et al., 2007; Yu et al., 2010). Other H_4_R antagonists with different chemical structure also block this response (Cowart et al., 2008; Liu et al., 2008; Koenig et al., 2010; Shin et al., 2012; Savall et al., 2014). The data then clearly support the role of JNJ 77777120 as an antagonist for this effect. The compound reverses the effect of agonists, mimics the findings in animal lacking the H_4_R and the results are replicated by other antagonists. However, this cannot be generalized. For example JNJ 7777120 also has been shown to block substance P induced itch (Yamaura et al., 2009) and while is it appropriate to speculate that H_4_R activation is involved in substance P induced itch based on data in other models, it would take studies in H_4_R-deficient mice or with other structurally distinct antagonist to firmly conclude this.

**Table 2:** Effects of H_**4**_R-deficient mice in selected animal models^**1**^.

Disease models	Effect	Reference
Asthma	Decreased BAL eosinophils, macrophages, and lymphocytes Decreased IL-5, IL-4, IL-6, IL-17 levels Decreased antigen specific IgE and IgG_1_ levels	Dunford et al. (2006)
Dermatitis	Reduced inflammation in FITC-induced dermatitis	Cowden et al. (2010b)
Pruritus	Reduced histamine-induced scratching Reduced hapten-induced scratching	Dunford et al. (2007), Cowden et al. (2010b)
EAE	Increased clinical score, demyelination, and inflammation Decreased T_reg_, increased Th17 cells	del Rio et al. (2012)
Arthritis	Decreased severity score Decreased inflammation, pannus formation, cartilage and bone damage	Cowden et al. (2014)


^1^BAL, bronchoalveolar lavage; EAE, experimental autoimmune encephalomyelitis.

Some of the *in vivo* data also yield insight as to the role of functional selectivity at the H_4_R. In the models of pruritus, asthma, and dermatitis the phenotype is the same between JNJ 7777120-treated and H_4_R-deficient mice (Dunford et al., 2006, 2007; Cowden et al., 2010b). This suggests that if the induction of β-arrestin shown by Rosethorne and Charlton (2011) occurs in mice (currently functional selectivity has only been studied for the human receptor), it has little impact on the activity of the compound in these models. For if JNJ 7777120-induced arrestin pathways are anti-inflammatory then they would not exist in the receptor deficient animals. Once again the most likely explanation is that the compound functions as an antagonist of the receptor. This does not mean, however, that the possibility of functional selectivity should be dismissed and it may suggest that antagonists could be created that only block a subset of the pathways activated by the receptor.

## Function

Much of the original expression data obtained with the H_4_R pointed to expression on cells involved in immune responses. In conjunction with this, researchers took advantage that the ligand for this orphan receptor was histamine and thus searched the extensive literature on histamine to find functions mediated by histamine, but where the pharmacology did not clearly point to the H_1_R, H_2_R, or H_3_R. One example of this was in eosinophils. In Clark et al. (1975) showed that histamine could induce chemotaxis of human eosinophils at low concentrations, 10^-7^ and 10^-6^ M, but that at 10^-5^ M chemotaxis was reduced. They showed that neither mepyramine (an H_1_R antagonist) nor metiamide (an H_2_R antagonist) had any effect on the enhanced chemotaxis, but that metiamide reversed the inhibition of chemotaxis at 10^-5^ M. They concluded that the stimulation of chemotaxis by histamine was independent of H_1_R and H_2_R and in a later paper concluded that a third histamine receptor existed on eosinophils (this was prior to the discovery of the H_3_R; Clark et al., 1977). Raible et al. (1992) showed that histamine induced a calcium response in eosinophils and, as observed for chemotaxis, this effect was not inhibited by an H_1_R antagonist (mepyramine) or an H_2_R antagonist (cimetidine). The calcium response was inhibited by thioperamide which was described as an antagonist of the newly discovered H_3_R. In addition (R)-α-methylhistamine, an H_3_R agonist, induced a calcium response similar to that of histamine. However, the authors commented that the fact that (R)-α-methylhistamine was less potent than histamine suggested that this calcium response was not mediated by a classic H_3_R since at this receptor histamine is more potent than (R)-α-methylhistamine. In a later study Raible et al. (1994) confirm this unusual pharmacology with a series a different H_3_R antagonists and agonists. The identity of this novel histamine receptor remained a mystery (or was perhaps ignored) until the discovery of the gene for the H_4_R. It was noted that the pharmacological profile of the H_4_R was similar to the receptor described by Raible et al. (1994; Oda et al., 2000; Hough, 2001; Liu et al., 2001; Morse et al., 2001; Zhu et al., 2001; O’Reilly et al., 2002). In particular, burimamide is a weak H_4_R partial agonist (Zhu et al., 2001; Lim et al., 2005), while (R)-α-methylhistamine and *N*-methylhistamine are both full agonist (Nakamura et al., 2000; Oda et al., 2000; Liu et al., 2001; Morse et al., 2001; Zhu et al., 2001), but are less potent than histamine thus matching the conflicting pharmacology described by Raible et al. (1992, 1994). The role of the H_4_R in mediating histamine-induced eosinophil chemotaxis and calcium responses was confirmed once selective ligands became available. Selective H_4_R antagonists (e.g., JNJ 7777120) have been shown to block histamine-induced chemotaxis and calcium responses (Ling et al., 2004; Strakhova et al., 2009; Reher et al., 2012; Shin et al., 2012). Chemotaxis of eosinophils can also be studied indirectly by measuring shape-change related to actin reorganization that precedes chemotaxis (Sabroe et al., 1999). Histamine and selective H_4_R agonists such as 4-methylhistamine and others have been shown to induce eosinophil shape change and this can be blocked by H_4_R selective antagonists (Ling et al., 2004; Lim et al., 2005; Yu et al., 2010; Shin et al., 2012; Thurmond et al., 2014a).

The histamine-induced eosinophil shape change was used as a pharmacodynamics readout for a clinical study with JNJ 39758979 (Thurmond et al., 2014a). After oral dosing of the compound to healthy volunteers, blood samples were taken at various timepoints, stimulated with histamine and the shape change of the eosinophils was assessed. At doses and time points where the concentration of JNJ 39758979 was above 100 nM, a statistically significant inhibition was observed. This level of potency was similar to what when the compound was added to blood *in vitro* (Thurmond et al., 2014a). These results show that *in vivo* administration of an H_4_R antagonist in humans can have impact on eosinophil function.

The initial idea that a new histamine receptor was present on eosinophils came about because of differences in the pharmacology of various histamine ligands. Not only were H_1_R and H_2_R ligands ineffective, but the potency of H_3_R ligands was not as expected (Raible et al., 1994). Therefore, it is important to evaluate the pharmacological data with the H_4_R ligands carefully. First, it has been noted that the potency for eosinophil function of the various H_4_R ligands (either selective or non-selective) compared to histamine is in general agreement with their relative affinities thus supporting that this is in fact the H_4_R (Buckland et al., 2003; Ling et al., 2004; Lim et al., 2005; Yu et al., 2010; Reher et al., 2012). Furthermore, the K_i_ for antagonists can be calculated from studies where the EC_50_ of histamine is given using modifications of the Cheng and Prusoff (1973) equation. Ling et al. (2004) report IC_50_ values for JNJ 7777120 and thioperamide for eosinophil shape change and chemotaxis. One can use these values along with the concentration and EC_50_ for histamine to calculate K_i_ values of 5 and 4 nM for JNJ 7777120 and 26 and 26 nM for thioperamide for inhibiting histamine-induced eosinophil shape change and chemotaxis, respectively. These numbers are similar to the reported Ki values from recombinant systems for JNJ 7777120 (4 nM) and thioperamide (27 nM; Liu et al., 2001; Thurmond et al., 2004). Consistent with this Barnard et al. (2008) report an IC_50_ of JNJ 7777120 of 6 nM that corresponds to a calculated K_i_ value of 2 nM. However, Seifert et al. (2011) have stated incorrectly that the discrepancy in the IC_50_ values for JNJ 7777120 between the Ling et al. (2004) and Barnard et al. (2008) studies (300 versus 6 nM) support the notion of functional selectivity for the H_4_R in eosinophils. However, the authors fail to take into account the differences in EC_50_ for histamine 19 nM for Ling et al. (2004) and 150 nM for Barnard et al. (2008), which when properly accounted for yields almost identical K_i_ values for JNJ 7777120 (5 nM versus 2 nM). The differences in the EC_50_ for histamine between the two studies is most likely due to large differences in histamine response related to H_4_R levels between difference donors as reported by Yu et al. (2010).

The effect of JNJ 7777120 on the histamine calcium response in eosinophils yields the same conclusion with a reported K_i_ value of 1.3 nM (Reher et al., 2012). However, these same authors report a discrepancy in the K_i_ values for JNJ 7777120 with respect to chemotaxis. The calculated K_i_ for JNJ 7777120 was 9.8 nM when histamine was used to induce chemotaxis, but 1.6 nM when a selective H_4_R agonist was used, UR-PI376 (Reher et al., 2012). They use this data as “evidence for ligand-specific receptor conformation” as the title states. However, the authors fail to account for the role of the H_2_R in inhibiting chemotaxis. The K_i_ value of 9.8 nM is calculated using the EC_50_ of histamine (120 nM), but this is not a true EC_50_ since the dose response curve is made up of two components – one for the H_4_R response and one for the H_2_R response. Indeed when the authors block the H_2_R with famotidine, the EC_50_ of histamine is reduced to 39 nM. Recalculating the K_i_ for JNJ 7777120 using this EC_50_, which represents the true H_4_R response, yields a value of 3.2 nM that is completely consistent with the value when using the H_4_R-selective agonist and the value reported by Ling et al. (2004). Furthermore, this value is similar to the K_i_ value the authors report for the histamine-induced calcium response because there is no H_2_R contribution to the calcium signal as shown in the paper (Reher et al., 2012). Therefore, the effects seen by H_4_R ligands on eosinophil responses are entirely consistent with the known pharmacology of the H_4_R and no functional selectivity needs to be invoked.

Histamine is largely associated with mast cells since these cells store and secrete large amounts of histamine. Therefore, it was natural early to look for H_4_R effects in mast cells. One of the major functions of mast cells it to release inflammatory mediators such as histamine upon IgE-mediated degranulation. However, in mouse bone marrow-derived mast cells, neither genetic deficiency (mast cells taken from H_4_R-deficient mice) nor the H_3_R/H_4_R antagonist thioperamide had any effect on antigen-mediated degranulation (Hofstra et al., 2003). The H_4_R does appear to modulate degranulation indirectly by inducing upregulation of high affinity IgE receptors, FcεRI (Mirzahosseini et al., 2013). In addition to this, in human mast cells histamine and 4-methylhistamine on their own were able in induced degranulation and in both cases this could be blocked with JNJ 7777120 (Jemima et al., 2014). As for eosinophils, histamine acting via the H_4_R can induce increases in intracellular calcium. This histamine-induced calcium response is not present in mast cells from H_4_R-deficient mice and can be blocked by thioperamide or JNJ 7777120 (Hofstra et al., 2003; Thurmond et al., 2004; Jemima et al., 2014). H_4_R agonists can also induce calcium responses in these cells (Yu et al., 2010; Jemima et al., 2014). Also similar to eosinophils, histamine-induced chemotaxis can be observed in mast cells. This effect was not observed with mast cells deficient in the H_4_R and can be blocked by antagonist of the H_4_R, but not the other histamine receptors (Hofstra et al., 2003; Thurmond et al., 2004, 2014a). As for the eosinophil data the IC_50_ values for H_4_R antagonist was consistent with measured values indicating that the effects seen are due to H_4_R antagonism (Thurmond et al., 2004, 2014a). Changes in cell shape related to chemotaxis can also be measured and blocked by H_4_R antagonists (Strakhova et al., 2009). Consistent with this, the H_4_R agonist 4-methylhistamine can induce chemotaxis and this effect is blocked by JNJ 7777120 (Lim et al., 2005). In addition, histamine has also been shown to potentiate the migration of human precursor mast cells to the chemoattractant CXCL12 (Godot et al., 2007). The histamine-induced migration of mast cells may be related to the accumulation of mast cells at sites of inflammation. It has been shown that inhalation of histamine by mice leads to an accumulation of subepithelial mast cells in the trachea and this was reversed upon pretreatment with JNJ 7777120 (Thurmond et al., 2004). The number of mast cells increases in the skin in mouse dermatitis models and *in vivo* treatment with JNJ 7777120 blocked this accumulation (Cowden et al., 2010b; Seike et al., 2010; Suwa et al., 2011; Matsushita et al., 2012; Ohsawa and Hirasawa, 2012). In addition to inducing chemotaxis, the H_4_R also enhances inflammatory mediator production from mast cells. In mouse mast cells, histamine and 4-methylhistamine were both able to induce IL-6 production on their own and potentiate the IL-6 production driven by LPS stimulation. These effects were mediated via the H_4_R since they could be blocked by H_4_R antagonists and were not present in H_4_R-deficient mice (Desai and Thurmond, 2011). In human mast cells the H_4_R mediated the release of leukotrienes, cytokines, and chemokines (Jemima et al., 2014).

The effects on mast cells and eosinophils pointed to allergic diseases such atopic dermatitis and asthma. The FITC-induced dermatitis model in mice is a contact dermatitis model, but unlike other models is characterized by eosinophilia. Mice deficient in the H_4_R, have reduced inflammation in this model as judged by a reduction in inflammatory cytokines and chemokines in the skin and a reduction in swelling at the site of FITC challenge (the ear; Cowden et al., 2010b). Several different H_4_R antagonists also show the same effect (Cowden et al., 2010b; Thurmond et al., 2014a). At the site of FITC application there is a increase in both the number of eosinophils and mast cells in the skin. Treatment of the mice systemically with JNJ 7777120 was able to reduce, and for mast cells completely block, this increase (Cowden et al., 2010b). The H_4_R does no play a role in all acute dermatitis models. For example, no effect was observed with H_4_R antagonists in an acute canine atopic dermatitis model or when 2,4-dinitrochlorobenzene or toluene-2,4-diisocyanate were used as the hapten in mice (Rossbach et al., 2009; Baeumer et al., 2011). The same lack of effect was reported with JNJ 7777120 in an acute model using the hapten 2,4,6-trinitro-1-chlorobenzene (TNCB; Seike et al., 2010). However, when TNCB was given chronically the administration of JNJ 7777120 reduced the inflammation, the levels of inflammatory cytokines, and the number of mast cells and eosinophils in the skin (Seike et al., 2010; Suwa et al., 2011; Matsushita et al., 2012). A reduction in inflammation by JNJ 7777120 was also observed in the NC/Nga mouse model of chronic allergic dermatitis (Ohsawa and Hirasawa, 2012). One explanation for the disconnect between some of the acute and chronic hapten models may be accounted for by differences in the cell types involved in the inflammation, i.e., Th1 versus Th2 cells.

Another disease typically associated with eosinophils and mast cells is asthma. The lack of effect for treating asthma with antagonists that target the H_1_R and H_2_R has led many to question the role of histamine in the disease (Thurmond et al., 2008). However, it is possible that histamine acts via the H_4_R to drive some of the pathophysiology of asthma. In support for a role of the H_4_R in asthma, polymorphisms in the gene have been associated with infection-induce asthma (Simon et al., 2012). In addition H_4_R-deficient mice are protected in a mouse asthma model (Dunford et al., 2006). These mice have fewer eosinophils in bronchoalveolar lavage fluid, reduced inflammatory cytokines, and decreased antigen specific IgE and IgG1 levels after allergen challenge (Dunford et al., 2006). Treatment with the H_4_R antagonists JNJ 7777120, JNJ 10191584, or JNJ 39758979 during allergen challenge also reduced the eosinophilia (Dunford et al., 2006; Cowden et al., 2010a; Beermann et al., 2012; Neumann et al., 2013; Thurmond et al., 2014a). Improvement in lung function has also been shown upon treatment with an H_4_R antagonist and in H_4_R-deficient mice (Cowden et al., 2010a; Hartwig et al., 2014). Similar effects have been reported in a guinea pig model, where JNJ 7777120 improved lung function, reduced inflammation, eosinophilia, and inflammatory mediator production in the lung after allergen challenge (Somma et al., 2013). All of these data point to a therapeutic potential for H_4_R antagonist for the treatment of asthma.

One unexpected finding in the mouse asthma models was that mast cells were not needed for the H_4_R response. Dunford et al. (2006) showed that the inhibition of lung eosinophilia and inflammatory cytokines levels by an H_4_R antagonist was still present in mice lacking mast cells. The model used in this case is known to be dependent on T cells and not mast cells (Komai et al., 2003), suggesting that T cells may be the main contributor to the H_4_R response. To support the effect of the H_4_R on T cells, JNJ 7777120 was dosed only around the sensitization in the asthma model when antigen specific T cells are being generated. As with the dosing at allergen challenge, treatment with the H_4_R antagonist at sensitization reduced the number of eosinophils and the inflammatory cytokines (Dunford et al., 2006). These results coupled with the fact that the H_4_R antagonists worked in models known to be T cell dependent suggested that the receptor was playing a role in the priming and activation of T cells. This effect is reflected in the reduction in Th2 cytokines such as IL-4, IL-5, and IL-13 in BAL fluid or upon restimulation of splenocytes or lymphocytes (Dunford et al., 2006; Cowden et al., 2010a; Somma et al., 2013; Hartwig et al., 2014). The activation of T cells may also be the primary target in the models of atopic dermatitis as reduction in Th2 cytokines have been reported in the skin after treatment with an H_4_R antagonist (Cowden et al., 2010b; Seike et al., 2010; Matsushita et al., 2012; Ohsawa and Hirasawa, 2012).

There are several possible mechanisms for the H_4_R effect on T cells. In both asthma and dermatitis models treatment with an H_4_R antagonist reduced the number of T cells at the site of inflammation (Cowden et al., 2010a; Mahapatra et al., 2014). The reduction may be a result of decreases in the production of chemokines (Cowden et al., 2010a,b) or direct effects of histamine as a chemoattractant for T cells (Bryce et al., 2006; Morgan et al., 2007). Expression of the H_4_R has been reported on human Th2 cells and H_4_R agonists increased the expression of IL-31 (Gutzmer et al., 2009). The effects on T cells may also be indirect as there is evidence that the H_4_R is involved in dendritic cell function and may drive the response in asthma. When H_4_R-deficient dendritic cells where used to polarize T cells *in vitro*, these T cells there not able to transfer disease in a mouse adoptive transfer asthma model (Hartwig et al., 2014). This was not the case when H_4_R-deficient T cells were used. In addition, wild-type T cells could not transfer disease in H_4_R-deficient mice suggesting that it is the H_4_R on other cells such as dendritic cells that are important for driving disease (Hartwig et al., 2014). One possible role for the H_4_R in dendritic cells is in mediating migration of the cells to the site of interaction with T cells, since it has been shown that the migration of antigen positive dendritic cells from the skin to the lymph node was reduced upon treatment with an H_4_R antagonist (Cowden et al., 2010b). As for T cells, these effects may be a result of H_4_R directly mediating migration via histamine acting a chemoattractant (Gutzmer et al., 2005; Damaj et al., 2007; Bäumer et al., 2008; Gschwandtner et al., 2010, 2011) or indirectly by reduction in chemokine production (Cowden et al., 2010a,b). The H_4_R may also impact activation of dendritic cells. Dendritic cells from H_4_R-deficient mice or treated *in vitro* with an H_4_R antagonists were defective in their ability to activate Th2 cells (Dunford et al., 2006). Similarly, when human monocyte derived dendritic cells were treated with JNJ 7777120 the levels of MHC and costimulatory molecules were reduced and these cells were unable to induce allergen-specific proliferation of T cells (Lundberg et al., 2011). In addition to effects on dendritic cell maturation, the H_4_R modulates cytokine and chemokine production by dendritic cells that may result in defective T cell activation (Gutzmer et al., 2005; Dunford et al., 2006; Dijkstra et al., 2008; Gschwandtner et al., 2010, 2011, 2012). Dendritic cells can be activated by endogenous danger signals action via toll-like receptors (TLRs). Activation of dendritic cells *in vitro* with TLR ligands leads to the production of cytokines and chemokines and this can be modulated by H_4_R antagonists (Gutzmer et al., 2005; Dunford et al., 2006; Dijkstra et al., 2008; Gschwandtner et al., 2010, 2011, 2012). *In vivo* H_4_R antagonist can inhibit LPS (a TLR ligand) induced production of inflammatory cytokines and this is also reduced in H_4_R-deficient mice (Cowden et al., 2013). This link between TLR and H_4_R may important driving asthmatic responses since it has been shown that H_4_R antagonists are only effective in mouse asthma models when LPS is present (Cowden et al., 2013).

The role for the H_4_R in T cells pointed to possible roles in other disease that are thought to be T cell mediated. In particular the autoimmune disease rheumatoid arthritis is thought to be driven in part by T cell responses as evidenced by the clinical efficacy of abatacept, that targets the activation of T cells, and the fact that there is a strong genetic association with the human leukocyte antigen (HLA)-DRB1 and the presentation of antigens (Raychaudhuri et al., 2012; Gizinski and Fox, 2014). Based on this, the role of the H_4_R in preclinical models of rheumatoid arthritis has been explored. H_4_R-deficient mice and mice treated with the H_4_R antagonist JNJ 28307474 were protected from disease in both a mouse collagen-induced arthritis and collagen antibody-induced arthritis model (Cowden et al., 2014). This was reflected in an improvement in disease severity score and in the histologic analysis of the joints. The anti-inflammatory effects can be observed either with dosing at the onset of disease or at the peak of disease activity. A second selective H_4_R antagonist, JNJ 39758979, has also shown activity in the collagen-induced arthritis model (Savall et al., 2014). However, the effect on T cells may not translate into efficacy in all autoimmune diseases. H_4_R-deficient mice or mice treated with an H_4_R antagonist had significantly worse disease in mouse experimental autoimmune encephalomyelitis models of multiple sclerosis (del Rio et al., 2012; Ballerini et al., 2013).

The collagen-induced arthritis model is known to be driven by the production of IL-17 from Th17 cells (Lubberts et al., 2001; Nakae et al., 2003; Lubberts et al., 2004). Treatment with the H_4_R antagonist in this model decreased the production of IL-17 from lymphocytes and reduced the number of IL-17 positive CD4 cells (Th17 cells) in the inguinal lymph node, but had no effect on the production of IFNγ. H_4_R antagonists also blocked the differentiation of Th17 cells *in vitro* and reduction in IL-17 production has also been reported in models of asthma and dermatitis (Dunford et al., 2006; Cowden et al., 2010b, 2014). As for the asthma model, the effects on T cells could be direct or indirect. For the collagen antibody-induced model, adoptive transfer of wild-type dendritic cells (splenic CD11c^+^ cells) restored disease in H_4_R-deficient animals suggesting that the H_4_R on antigen presenting cells was crucial for disease activity (Cowden et al., 2014). This is also suggested when Th17 development was studied directly *in vivo* using an adoptive transfer model. The H_4_R antagonist blocked the Th17 differentiation of transgenic T cells *in vivo*. In addition the transfer of wild-type transgenic T cells into H_4_R-deficient host mice also resulted in a reduction in Th17 cells supporting the conclusion that the H_4_R on host antigen presenting cells is important (Cowden et al., 2014). However, this may not be the complete story as transfer of H_4_R-deficient transgenic T cells into wild-type host also lead to a reduction in Th17 cells indicating that the H_4_R on T cells is also important. Expression the H_4_R has been reported on human and mouse Th17 cells (Mommert et al., 2012; Cowden et al., 2014). In isolated human Th17 cells H_4_R agonist increased the production of IL-17 and this was reduced by an H_4_R antagonist suggesting that the receptor can have a direct effect on Th17 cells (Mommert et al., 2012).

Pruritus is another process that has long been associated with histamine (for a review see Thurmond et al., 2014b). In fact, injection of histamine into the skin of humans causes the sensation of itching. Antihistamines that target the H_1_R have exhibited efficacy in reducing itch in a number of conditions such as acute urticaria and allergic rhinitis. However, the itch associated with other pruritic diseases such as atopic dermatitis is not well-controlled by these drugs. The discovery of the H_4_R prompted the exploration as to whether this receptor could be responsible for pruritic responses not driven by the H_1_R. Injection of histamine into the skin of mice causes a scratching response. This response can be inhibited by the administration of H_4_R antagonists (Bell et al., 2004; Dunford et al., 2007; Yamaura et al., 2009; Shin et al., 2012; Savall et al., 2014). Histamine-induced scratching is also reduced in H_4_R-deficient mice (Dunford et al., 2007). Injection of H_4_R agonists can induce scratching and this is inhibited with H_4_R antagonists or in H_4_R-deficient mice (Dunford et al., 2007; Cowart et al., 2008; Liu et al., 2008; Koenig et al., 2010; Yu et al., 2010). H_4_R antagonists can also inhibit scratching in mice induced by substance P, haptens, and in models of dermatitis (Rossbach et al., 2009; Yamaura et al., 2009; Cowden et al., 2010b; Suwa et al., 2011; Ohsawa and Hirasawa, 2012). These data have prompted the study of the anti-pruritic effects of H_4_R antagonists in humans.

## Clinical Data

There have only been a few reports of clinical data with H_4_R antagonists. The compound with the most published information is JNJ 39758979. JNJ 39758979 is a potent and selective H_4_R antagonist that has efficacy in preclinical models of pruritus, dermatitis, asthma, and arthritis (Savall et al., 2014; Thurmond et al., 2014a). This H_4_R antagonist has been used to explore the role of the receptor in mediating histamine-induced pruritus in humans (NCT01068223). Over three periods subjects received either a single dose of JNJ 39758979, the H_1_R antagonist cetirizine, or placebo. At 2 and 6 h after dosing, histamine was injected intradermally into the skin of the forearm of each subject and the pruritic response was assessed by having the subject rate the itch sensation on 1–10 scale over a 10 min period (Kollmeier et al., 2014). JNJ 39758979 reduced the itch sensation induced by histamine at both 2 and 6 h after dosing, whereas placebo had no effects. Cetirizine was used as a positive control and it reduced the pruritic response at 6 h post-dose as expected. This data validates the preclinical findings in mice and shows that the H_4_R is involved in mediating pruritic responses in humans. This suggests that H_4_R antagonists will have utility in humans in treating pruritic conditions known to be mediated by histamine such as acute urticaria and allergic rhinitis. Of potential interest is the drug alcaftadine that is a topical ophthalmic solution indicated for the prevention of itching associated with allergic conjunctivitis. This compound has weak activity at the H_4_R as well as being a potent H_1_R antagonist and this differentiates it from other topical treatments such as olopatadine that has no H_4_R activity (Gallois-Bernos and Thurmond, 2012). Preclinical data indicate that the combination of H_4_R and H_1_R may yield better efficacy against pruritus than an H_1_R alone (Dunford et al., 2007; Nakano et al., 2009; Rossbach et al., 2009; Cowden et al., 2010b; Ohsawa and Hirasawa, 2012). This may also be true in humans as an analysis across two clinical efficacy studies indicated that alcaftadine exhibited greater efficacy in reducing ocular itch compared to olopatadine after conjunctival allergen challenge (McLaurin et al., 2014).

One pruritic condition where the itch is not well controlled by H_1_R antagonists is atopic dermatitis, which is a common inflammatory pruritic skin disease. Pruritus is one of the most common and characteristic symptoms of atopic dermatitis (Williams, 2005). Several clinical trials have been conducted to evaluate the efficacy of H_1_R antagonists for the reduction of pruritus associated with atopic dermatitis, with limited evidence for efficacy (Klein and Clark, 1999; Akdis et al., 2006; Saeki et al., 2009). These observations prompted the study of H_4_R antagonists in preclinical dermatology models and indicated that such antagonists could be efficacious (Cowden et al., 2010b; Seike et al., 2010; Suwa et al., 2011; Matsushita et al., 2012; Ohsawa and Hirasawa, 2012). Evidence that H_4_R can be efficacious against atopic dermatitis in humans has been provided in the clinical with JNJ 39758979. A phase 2a study in adults with moderate atopic dermatitis compared two doses of JNJ 39758979 versus placebo (Murata et al., 2015). Unfortunately, the study was terminated before all subjects completed the treatment period due to two cases of agranulocytosis that was most likely related to reactive metabolites of the compound and not to H_4_R antagonism (Murata et al., 2015). *Post hoc* analysis indicated evidence of efficacy. The primary efficacy was assessed at week 6 using eczema area and severity index (EASI) scores. There was a numerical reduction in the EASI score for both 100 and 300 mg JNJ 39758979 compared to placebo, but these changes were not statically significant. The EASI score does not include any direct measurements of pruritus, which is the most common and characteristic symptom of atopic dermatitis, and therefore several secondary endpoints were include to assess pruritus. Across all of these endpoints there was a strong and nominally statistically significant reduction in the pruritus sensed by patients on JNJ 39758979 (Murata et al., 2015). Therefore although there are caveats with the interpretation of the results and issues with the safety of JNJ 39758979, it appears that other safer H_4_R antagonists could have utility in the treatment of atopic dermatitis especially with respect to reducing pruritus.

In addition to the published studies a study with JNJ 39758979 in patients with persistent asthma (NCT00946569) has been completed, but no data has been reported. In addition to this other H_4_R antagonists have been reported to be in the clinic such as UR-63325, PF-3893787, and toreforant (JNJ 38518168), the first H_4_R antagonist with a generic name. ClinicalTrials.gov indicates that UR-63325 has completed a nasal allergen challenge study in patients with allergic rhinitis (NCT01260753) and PF-3893787 a bronchial allergen challenge study in patients with asthma (NCT00856687), but no results have been reported. Toreforant has completed efficacy studies in patients with rheumatoid arthritis (NCT01679951, NCT00941707, and NCT01862224). Results for these studies have not been reported, but it was noted that a dose range finding study in rheumatoid arthritis (NCT01679951) was terminated for lack of efficacy. Studies with toreforant in patients with asthma (NCT01823016) and psoriasis (NCT02295865) were ongoing as of February 2015.

## Conclusion

The story of the H_4_R provides an excellent case study for the deorphanization of a novel receptor and the translation of this into clinical efficacy in humans. The gene for the receptor was discovered via genomic homology searches and reverse pharmacology led to the identification of its role in immune and pruritic responses. This work has now resulted in the first reports of clinical efficacy for H_4_R antagonists and point to the potential of these ligands as future drugs for the treatment of a variety of indications.

## Conflict of Interest Statement

Author is an employee and stock holder of Johnson and Johnson, the parent company of Janssen Research & Development.
